# Scalable Precursor-Assisted Synthesis of a High Voltage LiNi_y_Co_1−y_PO_4_ Cathode for Li-Ion Batteries

**DOI:** 10.3390/nano13243156

**Published:** 2023-12-16

**Authors:** Mobinul Islam, Ghulam Ali, Muhammad Faizan, Daseul Han, Basit Ali, Sua Yun, Haseeb Ahmad, Kyung-Wan Nam

**Affiliations:** 1 Department of Energy & Materials Engineering, Dongguk University, Seoul 04620, Republic of Korea; mobin85@dongguk.edu (M.I.); muhammadfaizan@dgu.ac.kr (M.F.); endend941206@gmail.com (D.H.);; 2 U.S.-Pakistan Center for Advanced Studies in Energy, National University of Sciences and Technology (NUST), Islamabad 44000, Pakistan; ali@uspcase.nust.edu.pk (G.A.);

**Keywords:** high-voltage cathode, Li-ion battery, hydroxide precursor, X-ray absorption spectroscopy

## Abstract

A solid-solution cathode of LiCoPO_4_-LiNiPO_4_ was investigated as a potential candidate for use with the Li_4_Ti_5_O_12_ (LTO) anode in Li-ion batteries. A pre-synthesized nickel–cobalt hydroxide precursor is mixed with lithium and phosphate sources by wet ball milling, which results in the final product, LiNi_y_Co_1−y_PO_4_ (LNCP) by subsequent heat treatment. Crystal structure and morphology of the product were analyzed by X-ray powder diffraction (XRD), X-ray photoelectron spectroscopy (XPS), and scanning electron microscopy (SEM). Its XRD patterns show that LNCP is primarily a single-phase compound and has olivine-type XRD patterns similar to its parent compounds, LiCoPO_4_ and LiNiPO_4_. Synchrotron X-ray absorption spectroscopy (XAS) analysis, however, indicates that Ni doping in LiCoPO_4_ is unfavorable because Ni^2+^ is not actively involved in the electrochemical reaction. Consequently, it reduces the charge storage capability of the LNCP cathode. Additionally, ex situ XRD analysis of cycled electrodes confirms the formation of the electrochemically inactive rock salt-type NiO phase. The discharge capacity of the LNCP cathode is entirely associated with the Co^3+^/Co^2+^ redox couple. The electrochemical evaluation demonstrated that the LNCP cathode paired with the LTO anode produced a 3.12 V battery with an energy density of 184 Wh kg^−1^ based on the cathode mass.

## 1. Introduction

Demand for lithium-ion batteries (LIBs) has been increasing rapidly in portable electronics, electric vehicles, and other industries [1,2,3]. The rapid progression of LIB technology spurred an immediate demand for novel high-energy-density systems. A great deal of work is being put into exploring novel materials that can be used as the cathode for high-voltage LIBs. The first commercialized cathode LiCoO_2_ has a high operating voltage (~3.9 V) [4]. However, LiCoO_2_ has been gradually replaced by other commercialized cathode materials, such as spinel LiMn_2_O_4_, olivine LiFePO_4_, and layered-type LiNi_1/3_Mn_1/3_Co_1/3_O_2_ (NCM111) during the continuous development of LIBs owing to the high cost of Co resources [1,5]. Introducing redox couples with high redox potentials, such as Co^2+^/Co^3+^, Ni^2+^/Ni^4+^ or V^3+^/V^4+^/V^5+^, can further boost the voltage and energy density [6]. For example, LiNi_0.5_Mn_1.5_O_4_ demonstrates dramatically increased redox potential from 4.0 to 4.7 V by a partial replacement of Mn with Ni in spinel LiMn_2_O_4_ lattice [5]. In the case of layered LiNi_x_Mn_y_Co_z_O_2_ chemistry, where x:y:z = 5:3:2 (NMC532), 6:2:2 (NMC622), 8:1:1 (NMC811), the higher Ni fraction translates into the increased energy density, boosting research attention on Ni-rich layered oxides [7,8]. Alternatively, the olivine family LiMPO_4_ (M = Fe, Mn, Co) cathodes have become the subject of intensive research thanks to their remarkable thermal stability attributed to the strong covalent P–O bond within the polyanionic structure. Typically, oxide atoms are packed in a slightly distorted hexagonal close packing (HCP) in an olivine structure where half of the octahedral sites contain Li^+^ and M^2+^ cations, while 1/8 of the tetrahedral sites contain P^5+^ cations, which is a hexagonal analogue of spinel. The MO_6_ octahedrons share four corners in the bc plane and are cross-linked by PO_4_ groups along the ‘a’ axis. The Li ions reside inside rows of edge-shared LiO_6_ octahedra aligned along the ‘a’ axis. Li^+^ ions are occupied by the three-dimensional network of perpendicular channels along the [010]– and [001]– direction. Having this network plays a crucial role in lithium-ion mobility, which makes olivine a viable cathode material [9]. Among them, LiFePO_4_ is the pioneer, reported in 1997 by Goodenough and co-workers [10]. It has been extensively investigated and successfully commercialized due to its favorable electrochemical properties, exceptional safety, abundance of Fe resources, and environmental friendliness. In addition, LiCoPO_4_ and LiNiPO_4_ also emerged as promising cathode materials on the pathway to future high-voltage (5 V) batteries due to their high operating voltage ~4.8 V and ~5.1 V, respectively, which translate to theoretical energy densities of 801 and 862 Wh kg^−1^ [5]. Nevertheless, the sluggish kinetics of the electronic and lithium-ion transport for these cathodes pose a major constraint to their development. Numerous modifications have been adapted to improve the Li extraction/insertion kinetics and electronic conductivity, including morphology control [11,12], metal ion doping [13], metal oxide coating [14,15,16], and carbon coating [17,18]. Manickam Minakshi et al. have conducted several studies on olivine cathodes [19,20,21,22,23]. A LiCoPO_4_/C nanocomposite with controlled morphology was synthesized using solid-state fusion by introducing a second phase (Co_2_P) in an inert atmosphere and utilizing Super P for amorphous carbon coating [19]. A LiCo_1/3_Mn_1/3_Ni_1/3_PO_4_ cathode, synthesized by sol-gel (SG) and solid-state methods, demonstrated superior performance in aqueous batteries with even microparticle distribution aided by PVP in the SG process [20]. Beyond these approaches, the overall electrochemical performance of any olivine-type materials can be enhanced via solid solutions. Basically, among all the cationic redox couples, Ni^+2/+3/+4^ is the most significant contributor to boosting the energy density of cathode materials, as demonstrated in the Ni-rich layered oxides [8]. However, it is difficult to synthesize pure phase LiNiPO_4_ due to the segregation of the Li_4_P_2_O_7_ and Ni_3_P impurity phases [23]. Alternatively, Ni doping is often widely adopted to influence the structural modification and electrochemical activity of other phospho-olivine cathodes, LiMnPO_4_ (M = Fe, Mn, and Co). Y. Ge et al. [24] have attributed the Ni doping to reducing the particle size of LiFePO_4_. Y. Liu et al. [25] stated that doped Ni can enhance the crystallinity of LiFePO_4_, as evidenced by XRD analysis. Y. Lu et al. [26] stated that doping nickel in LiFePO_4_/C composites strengthens the P–O bond, makes the structure more stable, and lowers the cathode particles and charge transfer resistance. The theoretical calculation predicted that Ni doping in LiMnPO_4_ could enhance the insertion potential of Li and reduce activation barriers that inhibit Li diffusion [27]. M. Minakshi and S. Kandhasamy [28] demonstrated improved voltage and capacity in LiMnPO_4_ with Ni substitutions. D. Shanmukaraj et al. reported that substitution of Ni in LiCoPO_4_ strengthens the P–O and M–O bonds [29]. Therefore, LiNiPO_4_–LiCoPO_4_ solid solutions were also investigated computationally [30] and experimentally [31,32,33,34] as potential cathodes for use in Li-ion batteries. S. T. Rommel et al. [34] have reported the synthesis of the LiNi_1−y_Co_y_PO_4_ (y = 0.25, 0.33, 0.66, 1.0) and observed a stabilization effect of cycle life at an optimum Ni content of 33%.

Various methods have been developed to synthesize those olivine-type LIB cathodes, including co-precipitation [29], sol-gel [32], hydrothermal [35], microwave [36], solid-state [16], supercritical fluid [11,37], polyol [38], and spray pyrolysis [33]. Since many of the available Co and Ni precursors are susceptible to form impurities, such as Co metal, CoO, Ni_3_P, and Li_3_PO_4_, most of the synthesis routes reported cannot be scaled up or require complicated heat-treatment steps to achieve pure stoichiometric LiMPO_4_ (M = Ni, Co) [16,39]. For example, NH_4_CoPO_4_ nanoplates can be used for LiCoPO_4_ preparation [40]. However, multiple heat treatments in both an air and an inert atmosphere were needed to make sure that the LiCoPO_4_ was stoichiometric because there is a possibility of cobalt metal impurities being formed by the decomposition of NH_4_CoPO_4_. In general, for synthesizing Ni-containing layered cathode materials that are successfully commercialized, the routine procedure entails two steps: precursors are synthesized through co-precipitation reactions, followed by their lithiation through solid-state reactions at high temperatures [41]. In the battery industry, co-precipitation is advantageous because it can incorporate various raw materials, such as chlorides, sulfates, nitrate salts, and organic anions. Carbonate and hydroxide co-precipitations are the most popular methods to prepare the precursors for the layered-type cathodes. Nevertheless, the precursor-assisted synthesis received much less attention for preparing olivine-type LiMPO_4_ (M = Ni, Co) cathodes [42]. In addition, a post-mortem study of the cycled electrodes is essential to reveal the charge storage mechanism of electrode materials. By analyzing dQ/dV (differential capacity) vs. voltage curves, only one study attempted to unravel the electrochemical behavior of LiNiPO_4_–LiCoPO_4_ solid-solution cathodes [34]. X-ray absorption spectroscopy (XAS) is considered one of the best tools in the scientific community for studying the electrochemical processes in battery materials. Its main important characteristics include (i) its element specificity, enabling a particular element to be studied by concentrating on its K (or, in some cases, L) absorption edge; (ii) the flexibility to customize it to different sites (such as Co and P in LiCoPO_4_), providing complementary information on the same compound. The XAS technique can track changes in oxidation states and local environmental conditions of metal atoms in battery materials. When X-rays are exposed to the target sample, a 1s photoelectron is excited into low-lying unoccupied states of the central atom at the K-edge, resulting in a normalized absorbance at specific energy [43].

On the anode side, a safety concern associated with Li dendrite formation in conventional graphitic anodes has sparked researchers to look at alternatives, such as Li_4_Ti_5_O_12_ (LTO). Lithium dendrite growth risk and solid electrolyte interface (SEI) formation can ultimately be elevated in LTO due to its lower-lying energy states of Ti^3+/4+^ redox couple than the LUMO (lowest unoccupied molecular orbital) level of electrolytes, leading to improved safety [44]. However, the redox potential (1.55 V vs. Li/Li^+^) of LTO reduces the overall working voltage in practical cell configurations. LiFePO_4_/LTO and Li(Ni_1/3_Co_1/3_Mn_1/3_)O_2_ (NCM111)/LTO full-cell batteries, for example, have offered cell voltages of only 1.85 and 2.2 V, respectively [45,46]. For the LTO anode, therefore, it may be appropriate to use “5 V-class cathodes”—Li_2_CoPO_4_F, LiCoPO_4_, LiNiPO_4_, and a LiCoPO_4_-LiNiPO_4_ solid solution—to secure a high output potential.

In this paper, LiNi_y_Co_1−y_PO_4_ (y = 0.33) has been synthesized by a low-energy ball mill using a pre-synthesized nickel–cobalt hydroxide precursor. The precursor was prepared by a hydroxide co-precipitation method. In addition to offering the advantages of mass production, the proposed synthesis strategy provided better homogeneity in the product. Moreover, the charge compensation mechanism of LiNi_y_Co_1−y_PO_4_ (y = 0.33) solid solution is revealed using synchrotron X-ray absorption spectroscopy (XAS). To date, no publication has reported the precursor-based synthesis of the LiCoPO_4_-LiNiPO_4_ solid-solution cathode, including the electrochemical performance test in a full-cell configuration in combination with the charge compensation mechanism revelation in a LiPF_6_-containing electrolyte. Our study reveals that incorporation of Ni into the LiCoPO_4_ olivine system is not worthy, as the Ni^2+^/Ni^3+^ redox couple was not activated during electrochemical cycling, which adversely affected the cathode-specific capacity.

## 2. Experimental Section

### 2.1. Synthesis

The precursor, [Ni_0.33_Co_0.67_](OH)_2_, was synthesized by co-precipitation method. The starting materials, NiSO_4_∙6H_2_O (Kanto Co., Tokyo, Japan) and CoSO_4_∙6H_2_O (Kanto Co., Tokyo, Japan) aqueous solutions, were pumped into a continuously stirred tank reactor (CSTR, 4 L) under a N_2_ atmosphere. The reactor was also simultaneously filled with NaOH solution (aq) in addition to the desired amount of NH_4_OH solution (aq) to act as a chelate. We carefully controlled the pH (11–12), temperature (60 °C), and stirring speed (1000 rpm) of the mixture in the reactor. After washing and filtering with distilled water, the resultant precursor powders were dried in a vacuum oven overnight. Finally, the obtained pink precursor powders were ball milled (at 150 rpm) with the ZrO_2_ media in ethanol for 12 h with Li_2_CO_3_, and NH_4_H_2_PO_4_. After the slurry was collected, it was dried in an oven at 80 °C for 12 h. The mixture was transferred to a vacuum-controlled box furnace equipped with flowing inert gas and calcined at over 700 °C at a heating rate of 2 °C min^−1^ for 8 h in a reductive (Ar 96% + H_2_ 4%) atmosphere to yield LiNi_0.33_Co_0.67_PO_4_, the final product. A solid-state approach was used to prepare the LTO anode. To achieve a uniform mixture, stoichiometric amounts of Li_2_CO_3_ (Sigma-Aldrich, Burlington, MA, USA, 99%) and TiO_2_ (Junsei Chemical, Tokyo, Japan, 98.5%) were mixed in a mortar with a pestle. We added 5% extra Li_2_CO_3_ (wt.%) to the mixture to compensate for the possibility of Li vaporization during the calcination process. For homogeneous mixing, the mixture was ground repeatedly and then pressed into pellets at 300 bars by using a hydraulic press (Daehatech, Busan, Republic of Korea, HP-1B). As-prepared pellets were placed in a box furnace (MTI corporation, Richmond, CA, USA) and calcined at two stages with different temperatures, 850 and 950 °C, for 12 h each in air with an intermediate grinding step. The crystal structure and phase purity of the as-synthesized LTO anode were confirmed by X-ray diffraction analysis. The morphology of the LTO sample (inspected by Field emission-scanning electron microscopy, Fe-SEM) was micrometer-sized particles with an average diameter (primary particle) of 1.81 μm, as published in our previous research [47,48].

### 2.2. Electrochemical Measurements

We made the slurry of LNCP cathode containing the synthesized active material, carbon black (Super P), and polyvinylidene fluoride (PVDF) binder (Sigma-Aldrich, Burlington, MA, USA) with a weight ratio of 8:1:1 in N-methyl-2-pyrrolidone (NMP). The mixed slurry was cast onto an Al foil current collector. The active material loading of cathode used for this study was 2.8 mg cm^−2^. Before the electrode was used, it was dried in a vacuum oven at 80 °C for 12 h. The prepared working electrode was assembled into coin-type half cells (CR2032) in combination with a Li counter electrode, a polypropylene separator, and an electrolyte composed of 1 M LiPF_6_ in ethylene carbonate and ethyl methyl carbonate (3:7 volume ratio).

More details about structural characterization, LTO slurry making, full-cell assembly, electrochemical testing, ex situ sample preparation, and XAS measurement are described in Supplementary Materials.

## 3. Results and Discussion

**Figure 1:**
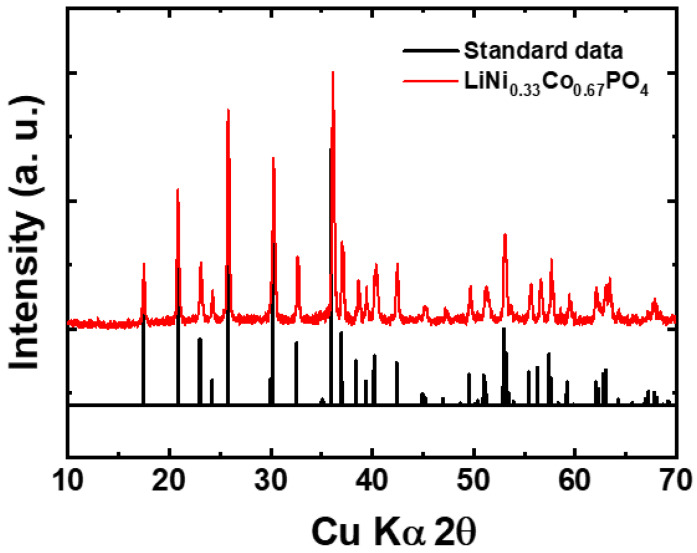
XRD pattern for LNCP powder after calcined at 700 °C.

The XRD result of LiNi_0.33_Co_0.67_PO_4_ (LNCP) material in Figure 1 exhibits well-defined diffraction peaks, matching with the standard data (ICSDS 00-000-1809) having the orthorhombic olivine structure with a *Pnma* space group. The sharp peaks reveal the high crystallinity of the material. A previous study observed lithium phosphate (Li_3_PO_4_) impurity phase formation when Mn is doped at the Co site [14]. Unlike this, no impurity peak was observed after the incorporation of the Ni at the Co site in the LiCoPO_4_ structure. The Ni_3_P is readily formed as a side phase during the synthesis of the LiNiPO_4_ material [32]. Herein, by maintaining a reductive atmosphere during heat treatment, Ni^2+^ and Co^2+^ are prevented from oxidizing into Ni^3+^ and Co^3+^, respectively, assuring that samples are impurity-free. Therefore, the XRD pattern signifies the formation of single-phase material.

**Figure 2:**
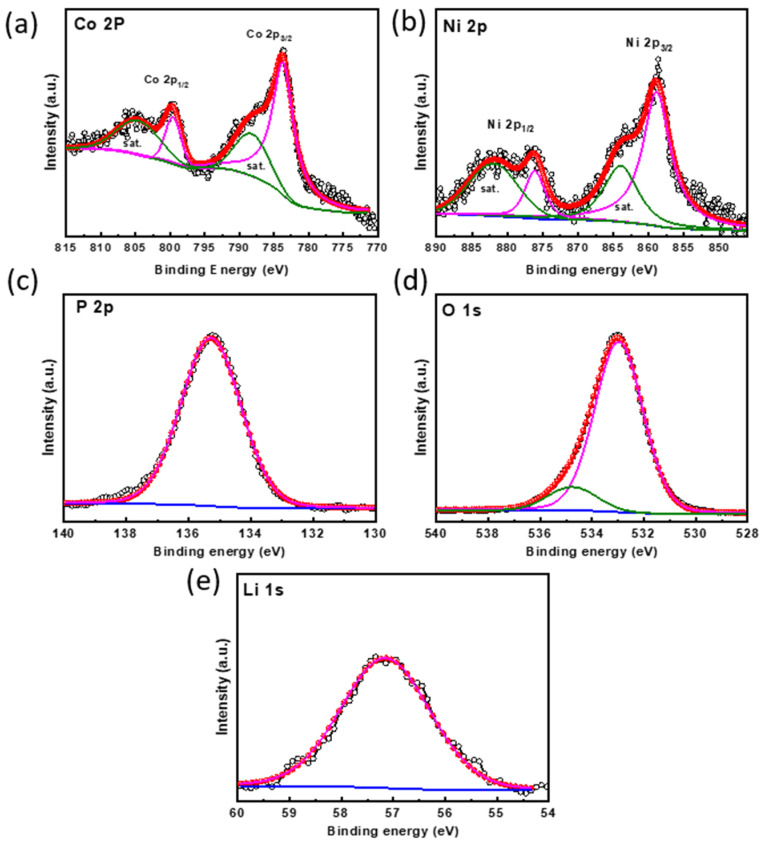
The high-resolution XPS spectra of (**a**) Co 2p, (**b**) Ni 2p, (**c**) P 2p, (**d**) O 1s, and (**e**) Li 1s in the LNCP powder.

The surface composition and valence state of the elements in the LNCP cathode were examined by X-ray photoelectron spectrometry (XPS). In Figure 2a, the Co 2p spectrum displays a typical spin-orbital split (2p_3/2_ and 2p_1/2_) with two shake-up satellite peaks at ~787.62 and 805.41 eV; the main peaks at 783.1 and 797.5 eV are attributed to Co^2+^ [49,50]. The Ni 2p region in Figure 2b exhibits two shake-up satellite peaks; the fitting peaks are located at the binding energies of 859 and 876.5 eV, indicating the oxidation states of Ni^2+^ [51]. The XPS spectrum for P 2p (Figure 2c) displays that the single binding energies peaked at 135.5 eV, which is assigned to the pentavalent P^5+^; this corresponds to the presence of the PO_4_^3−^ anion of LNCP [19]. Two peaks in the O 1s spectrum (Figure 2d) at ~530.13 and ~531.26 eV could be associated with metal phosphate and oxygen in chemically or physically adsorbed water molecules. The XPS spectrum of the Li 1s peak with binding energy 55.97 eV is shown in Figure 2e, confirming the presence of Li^+^ in LNCP.

**Figure 3:**
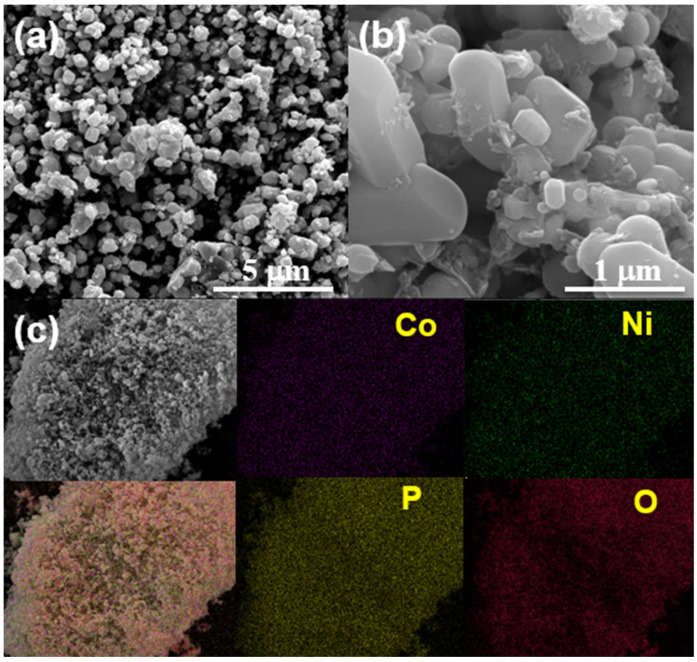
SEM micrographs at (**a**) low and (**b**) high resolutions; (**c**) SEM-EDS elemental mapping for LNCP.

The sample morphology was observed by FE-SEM, showing randomly distributed microparticles. The particles are loosely agglomerated without noticeable variation in their shape (Figure 3a). However, a closer inspection (Figure 3b) reveals two size distributions: small particles with a diameter range of about 200–400 nm appear on the surface of larger particles with an average size of 1–1.2 µm. It is evident that well-crystallized particles with a noticeable grain boundary and smooth surfaces are formed after calcination. During the electrochemical reaction, the loosely agglomerated particles allow the electrolyte to infiltrate into interparticle voids, ensuring proper utilization of the active material. Energy-dispersive X-ray spectroscopy (EDS) coupled with SEM was conducted to check the elemental distribution of the as-prepared LNCP cathode. In Figure 3c, SEM-EDS mapping results show that all elements, including Ni and Co, are homogeneously distributed within the sample. It is found that the atomic ratio of Ni and Co is 1:2 (Table S1).

**Figure 4:**
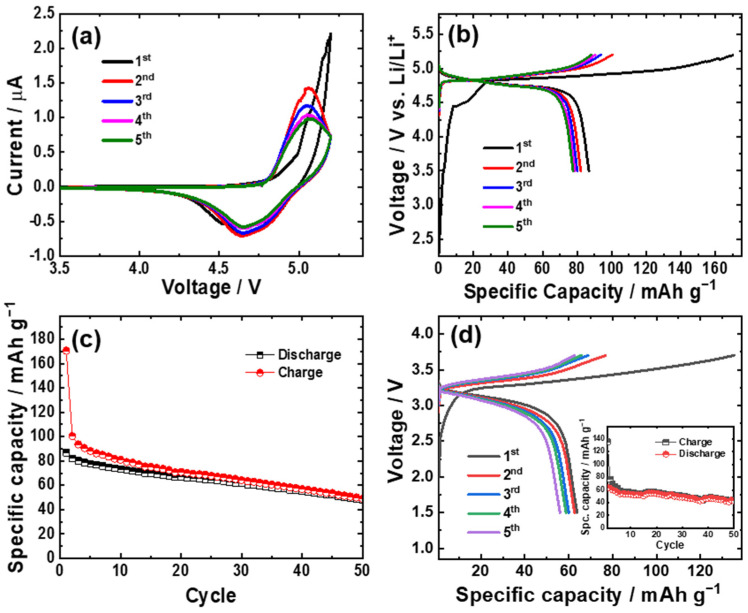
(**a**) Cyclic voltammetry at a scan rate of 0.1 mV s^−1^, (**b**) Galvanostatic voltage profiles, and (**c**) cycling performance of LNCP cathode material in half cell at 0.1 C-rate (1 C = 167 mAh g^−1^). (**d**) Voltage profile and cycling performance of LNCP–LTO full cell.

Figure 4a shows the electrochemical response of the LNCP cathode in the cyclic voltammetry (CV) test at a scan rate of 0.1 mV s^−1^ with a lithium foil counter electrode. The first anodic and cathodic sweep demonstrates the oxidation peaks at 5.19 V and 5.14 V and the reduction peaks at 4.66 V and 4.53 V, which were moved and merged on successive cycles at 5.05 V and 4.65 V, respectively. From the second cycle, the CV curve features almost the shape of the pure LiCoPO_4_ sample, as observed in a previous report [15,31]. Otherwise, the oxidation and reduction peaks of Co^2+^/Co^3+^ and Ni^2+^/Ni^3+^ might be so close to each other such that only single oxidation and reduction peaks appear in the following cycles. The respective charge and discharge curves of the LNCP cathode (Figure 4b) between 5.2 to 3.5 V over the first five cycles are coherent with the CV curves. As expected, divergent voltage plateaus emerged at variable cell potentials during the first and subsequent cycles. For lithium extraction, LiCoPO_4_ follows a two-phase mechanism for both aqueous [21] and non-aqueous [52] electrolytes. Our previous study demonstrated two distinguishable flat plateaus during the galvanostatic charge of LiCoPO_4_ indicative of a two-phase mechanism [53]. It is difficult to distinguish such plateaus from the second charge curve of the LNCP cathode here. Nevertheless, two anodic peaks in the dQ/dV plot (Figure S1) of the second cycle support a two-phase lithium extraction mechanism (Note: The extensive decomposition of the electrolyte associated with SEI layer formation on the electrode surface makes it difficult to discern anodic peaks on first cycle dQ/dV plot). The second-cycle charging and discharging capacities of the LCNP at 0.1 C (1 C = 167 mAh g^−1^) are 100.4 and 82 mAh g^−1^, respectively. The electrolyte decomposition and SEI layer formation at high voltages likely result in a substantial irreversible capacity loss and low (~51%) coulombic efficiency (CE) in the first cycle. However, there is a significant improvement in CE (i.e., 81.7, 83.1, 86.9, 88%) from the second to fifth cycles. After an initial activation period lasting ≈ 20 cycles, the CE reached over 95%, and the reversibility of the electrode is excellent for at least 50 cycles, highlighting that the target material can sustain a stable lifespan (Figure 4c). After 50 cycles, the discharge capacity values maintain ~48 mAh g^−1^. The electrolyte composition likely plays a significant role in LCNP capacity fading, which remains for further investigations. The oxidative decomposition of LiPF_6_-containing electrolytes was reported for the LiCoPO_4_ and other high-voltage materials [54,55]. Applying a sulfone-based electrolyte [56] or incorporating HF scavenger separators [57] into this high-voltage cathode would be favorable from the electrochemical performance standpoint. Generally, a LiNiPO_4_ cathode exhibits a flat plateau at approximately 5 V, corresponding to the Ni^3+^/Ni^2+^ redox couple [58]. The LNCP cathode shows a discharge voltage plateau at about 4.8 V, which is slightly lower considering the expected Ni^3+^/Ni^2+^ redox couple but is in good agreement with previous publications for the Co^3+^/Co^2+^ redox couple in LiCoPO_4_ [11,13]. When an LNCP cathode is combined with an LTO anode, this full cell displays a discharge plateau at 3.12 V in the voltage profile during the galvanostatic cycling test (Figure 4d). For the full cell, the described potential is not vs. Li/Li^+^; it is instead the potential difference between an LNCP cathode and an LTO anode. The practical capacity of as-synthesized LTO anode was 136 mAh g^−1^ at 0.1 C (1 C = 175 mAh g^−1^) in a half cell [48]. By considering the anode-to-cathode capacity ratio, the active mass of the assembled full-cell electrodes was regulated to ensure that the cathode material was fully utilized. The full cell shows a maximum specific discharge capacity of 64 mAh g^−1^ at 0.1 C (16.7 mA g^−1^), which corresponds to a specific energy of 184 Wh kg^−1^. These values are based on the weight of the cathodic active material rather than the entire device; the purpose is to highlight the material performance of LNCP. The energy density is determined from the integrals of the second discharge curve. The full cell retains a specific capacity value of 43 mAh g^−1^ after 50 cycles, as shown in Figure 4d (inset). It is important to note that the capacity retention of LNCP in a full cell is shown to be better than that of a half cell.

**Figure 5:**
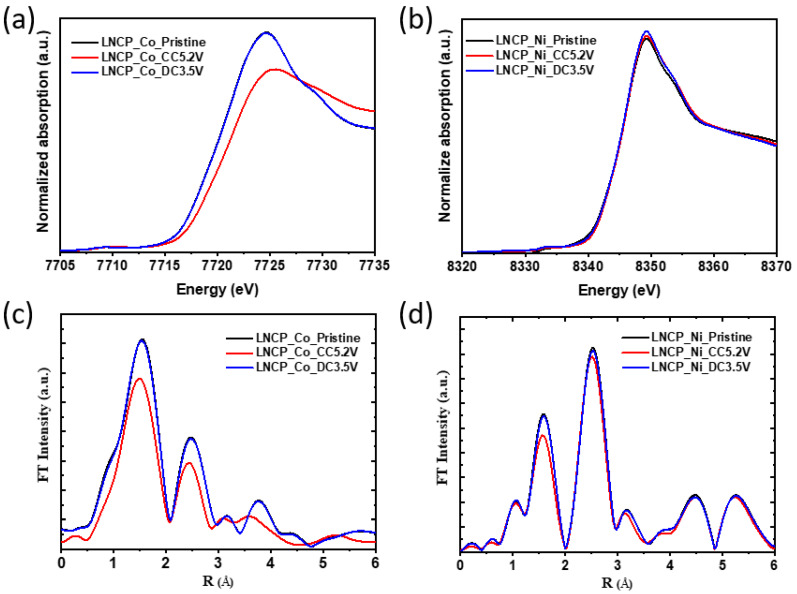
Ex situ analysis of LNCP electrodes during the first charge and discharge electrochemical cycle: (**a**) XANES and (**c**) corresponding EXAFS spectra at Co K-edge, and (**b**) XANES and (**d**) corresponding EXAFS spectra at Ni K-edge.

Despite the high working potential of the presented cathodic material, its low specific capacity results in relatively low specific energy compared to the cathodic materials (LiCoPO_4_) used in pairing with LTO [59,60]. To understand the causes of the low specific capacity, the charge compensation mechanism of the LNCP cathode was investigated using ex situ X-ray absorption spectroscopy (XAS). We examined the changes in the oxidation state of nickel and cobalt atoms throughout the electrochemical reactions using XANES. The ATHENA software package (version 0.9.26) was used to handle and process the XAS data [61]. There is an absorption edge that results from a dipole-allowed 1s → 4p transition, which is commonly called the white line, and the oxidation state change of the absorbing atom is described by its relative energy position shift. Figure 5a,b illustrates X-ray absorption near edge structure (XANES) spectra at the Co and Ni K-edges during charging to 5.2 V (full CC) and discharging to 3.5 V (full DCh). Delithiation (charge) leads to the entire edge shift of the Co K-edge toward higher energy, suggesting that Co^2+^ is being oxidized to Co^3+^. During lithiation (discharge), the Co K-edge XANES spectrum shows reversible edge shifts back to its pristine state, suggesting quite reversible Co^2+/3+^ redox reaction in the LNCP during charging and discharging. As opposed to this, the position of the Ni K-edge (Figure 5b) remains unchanged during charging and discharging. In light of this finding, it can be assured that the capacity contribution to the LNCP cathode is due to cobalt oxidation and reduction. The inactivity of the Ni redox couple explains the lower specific capacity of the target material. Figure 5c,d presents Fourier-transformed intensity of extended X-ray absorption fine structure (FT-EXAFS) spectra of LNCP showing interatomic distance changing behavior at the Co and Ni K edges. Typically, an FT peak represents the bond distance between the absorbing and backscattering atoms. At R = 1.5 Å, the first peak is attributed to the scattering of the nearest oxygen atom in the first coordination shell of the TM-O_6_ octahedron. At R = 2.45 Å, the second peak is associated with the scattering of the second nearest TM cation in the second coordination shell of the Co/Ni-TM_6_ hexagon. For the EXAFS process, the *k*-range was 3.0~11 Å^−1^ with a *k*-weight factor of 3. After complete charge to 5.2 V, the interatomic Co–O, and Co–TM distances decreased. Upon subsequent discharge, cobalt interatomic distances and peak intensities were completely restored to their original pristine (OCV) state, indicating the highly reversible local structural changes of cobalt in LNCP. Conversely, the Ni K-edge EXAFS spectra conform well to the NiO rock salt structure [62]. The Ni–O and Ni–TM interatomic distances remain unchanged, indicating that the inactive NiO rock salt phase adversely affects the charge storage capability in the LNCP cathode. Thus, as determined by XANES, the Ni^2+^ ions do not participate in electrochemical reactions, which is consistent with the interpretation of the FT-EXAFS results.

**Figure 6:**
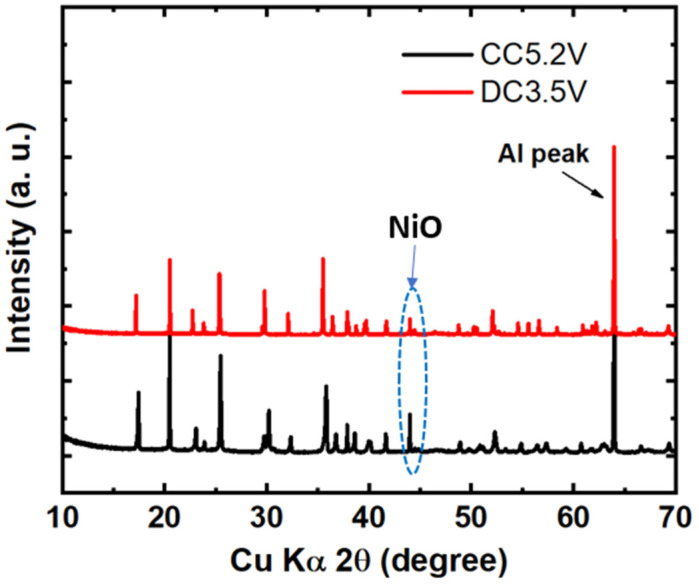
Ex situ XRD for fully charged and discharged LNCP electrodes in a 3.5–5.2 V voltage range.

After the charge/discharge cycle, ex situ X-ray diffraction (XRD) data were collected to obtain clues about the structural changes in the LNCP cathode as presented in Figure 6. It shows that all the main reflection peaks are maintained during charge–discharge cycles. Intense peaks are seen, which indicates suppression of amorphization of the LNCP cathode [63]. However, there is an additional peak at 2θ = 43.8° related to the NiO phase formation besides those for the LNCP material. This result confirms the detrimental rock salt phase (NiO) formation in the LNCP cathode [64]. Notably, S. M. Rommel et al. [34] also reported the inactivity of a Ni^2+^/Ni^3+^ redox couple in a LiCoPO_4_-LiNiPO_4_ solid solution cathode. However, the cause of the Ni content inactivity has not yet been identified. The present study has utilized a combination of ex situ XRD and synchrotron XAS techniques to reveal the underlying cause of this phenomenon. There is no doubt that the common polyanionic materials (LiMPO_4_) have structures that are generally stable with larger M cations (Fe^2+^, Co^2+^, Ni^2+^, and Mn^2+^, whose radii are 0.78, 0.72, 0.78, and 0.64 Å, respectively). The extremely small radius of the Ni^3+^ cation (0.56 Å) probably does not fit into the olivine structure’s octahedral environment or may cause strong structural distortion. Therefore, the electrochemical oxidation of the Ni^2+^ in LiNi_y_Co_1−y_PO_4_ material is most likely extremely difficult, at least under the standard conditions applied in this study.

## 4. Conclusions

A LiCoPO_4_-LiNiPO_4_ solid-solution cathode was synthesized using a pre-synthesized metal hydroxide precursor followed by wet ball milling with lithium (Li_2_CO_3_) and phosphate (NH_4_H_2_PO_4_) sources and subsequent heat treatment. The XRD result initially suggested that LiNi_y_Co_1−y_PO_4_ (y = 0.33) solid solutions were formed. The XPS results confirmed that in the pristine sample, the oxidation states of Co and Ni are +2. However, the results of synchrotron X-ray absorption spectroscopy (XAS) analysis of cycled electrodes indicate that Ni was not entirely doped into the structure of LiCoPO_4_. The cathode delivered low discharge capacity because the electrochemical activity of the Ni^2+^/Ni^3+^ redox couple was not activated due to the formation of electrochemically inactive rock salt-type NiO. Both cyclic voltammetry and discharge curves indicate that the LNCP cathode is driven by one redox couple, Co^3+^/Co^2+^, as confirmed by the XANES and EXAFS results. Therefore, substitution of Co^2+^ in LiCoPO_4_ by Ni^2+^ ion, in fact, negatively affects the cathode performance.

## Data Availability

All the data analyzed in this study are available on request.

## Supplementary Materials

The following supporting information can be downloaded at https://www.mdpi.com/article/10.3390/nano13243156/s1, Figure S1. The dQ/dV curves of LiNi_y_Co_1−y_PO_4_ cathode in a half cell at (a) initial and (b) second cycles. Figure S2. Voltage profile of the LNCP/graphite full cells: (a) without pre-lithiation and (b) with pre-lithiated anode. Table S1. SEM-EDS analysis result for the LNCP cathode.

## References

[1] Xu C., Dai Q., Gaines L., Hu M., Tukker A., Steubing B. (2020). Future material demand for automotive lithium-based batteries. Commun. Mater..

[2] Olivetti E.A., Ceder G., Gaustad G.G., Fu X. (2017). Lithium-ion battery supply chain considerations: Analysis of potential bottlenecks in critical metals. Joule.

[3] Liang Y., Zhao C.-Z., Yuan H., Chen Y., Zhang W., Huang J.-Q., Yu D., Liu Y., Titirici M.-M., Chueh Y.-L. (2019). A review of rechargeable batteries for portable electronic devices. InfoMat.

[4] Mizushima K., Jones P.C., Wiseman P.J., Goodenough J.B. (1980). Li_x_CoO_2_ (0 < x < −1): A new cathode material for batteries of high energy density, Mater. Res. Bull..

[5] Li W., Song B., Manthiram A. (2017). High-voltage positive electrode materials for lithium-ion batteries. Chem. Soc. Rev..

[6] Jeong G., Kim Y.-U., Kim H., Kim Y.-J., Sohn H.-J. (2011). Prospective materials and applications for Li secondary batteries. Energy Environ. Sci..

[7] Li W., Lee S., Manthiram A. (2020). High-Nickel NMA: A Cobalt-free alternative to NMC and NCA Cathodes for Lithium-ion Batteries. Adv. Mater..

[8] Mohamed N., Allam N.K. (2020). Recent advances in the design of cathode materials for Li-ion batteries. RSC Adv..

[9] Fisher C.A.J., Hart Prieto V.M., Islam M.S. (2008). Lithium battery materials LiMPO_4_ (M = Mn, Fe, Co, and Ni): Insights into defect association, transport mechanisms, and doping behavior. Chem. Mater..

[10] Padhi A.K., Nanjundaswamy K.S., Goodenough J.B. (1997). Phospho-olivines as positive-electrode materials for rechargeable lithium batteries. J. Electrochem. Soc..

[11] Truong Q.D., Devaraju M.K., Honma I. (2014). Benzylamine-directed growth of olivine-type LiMPO_4_ nanoplates by a supercritical ethanol process for lithium-ion batteries. J. Mater. Chem. A.

[12] Manzi J., Curcio M., Brutti S. (2015). Structural and morphological tuning of LiCoPO_4_ materials synthesized by solvo-thermal methods for Li-cell applications. Nanomaterials.

[13] Wang Y., Qiu J., Li M., Zhu X., Wen Y., Li B. (2022). One-Step Synthesis of LiCo_1−1.5x_Y_x_PO_4_@C cathode material for high-energy lithium-ion batteries. Materials.

[14] Örnek A., Can M., Yeşildağ A. (2016). Improving the cycle stability of LiCoPO_4_ nanocomposites as 4.8 V cathode: Stepwise or synchronous surface coating and Mn substitution. Mater. Charact..

[15] Eftekhari A. (2004). Surface modification of thin-film based LiCoPO_4_ 5 V cathode with metal oxide. J. Electrochem. Soc..

[16] Tolganbek N., Yerkinbekova Y., Kalybekkyzy S., Bakenov Z., Mentbayeva A. (2021). Current state of high voltage olivine structured LiMPO_4_ cathode materials for energy storage applications: A review. J. Alloys Compd..

[17] Kumar P.R., Rao V.M., Nageswararao B., Venkateswarlu M., Satyanarayana N. (2016). Enhanced electrochemical performance of carbon-coated LiMPO_4_ (M = Co and Ni) nanoparticles as cathodes for high-voltage lithium-ion battery. J. Solid State Electrochem..

[18] Örnek A., Can M., Yeşildağ A. (2018). Analysis of the effects of different carbon coating strategies on structure and electrochemical behavior of LiCoPO_4_ material as a high-voltage cathode electrode for lithium-ion batteries. Electrochim. Acta.

[19] Gangulibabu, Nallathamby K., Meyrick D., Minakshi M. (2013). Carbonate anion anion-controlled growth of LiCoPO_4_/C nanorods and its improved electrochemical behavior. Electrochim. Acta.

[20] Kandhasamy S., Pandey A., Minakshi M. (2012). Polyvinylpyrrolidone assisted sol-gel route LiCo_1/3_Mn_1/3_Ni_1/3_PO_4_ composite cathode for aqueous rechargeable battery. Electrochim. Acta.

[21] Minakshi M., Singh P., Sharma N., Blackford M., Ionescu M. (2011). Lithium extraction−insertion from/into LiCoPO_4_ in aqueous batteries. Ind. Eng. Chem. Res..

[22] Minakshi M., Singh P., Ralph D., Appadoo D., Blackford M., Ionescu M. (2012). Structural characteristics of olivine Li(Mg_0.5_Ni_0.5_)PO_4_ via TEM analysis. Ionics.

[23] Minakshi M., Singh P., Appadoo D., Martin D.E. (2011). Synthesis, and characterization of olivine LiNiPO_4_ for aqueous rechargeable battery. Electrochim. Acta.

[24] Ge Y., Yan X., Liu J., Zhang X., Wang J., He X., Wang R., Xie H. (2010). An optimized Ni doped LiFePO_4_/C nanocomposite with excellent rate performance. Electrochim. Acta.

[25] Liu Y., Gu Y.-J., Luo G.-Y., Chen Z.-L., Wu F.-Z., Dai X.-Y., Mai Y., Li J.-Q. (2020). Ni-doped LiFePO_4_/C as high-performance cathode composites for Li-ion batteries. Ceram. Int..

[26] Lu Y., Shi J., Guo Z., Tong Q., Huang W., Li B. (2009). Synthesis of LiFe_1−x_Ni_x_PO_4_/C composites and their electrochemical performance. J. Power Sources.

[27] Sgroi M.F., Lazzaroni R., Beljonne D., Pullini D. (2017). Doping LiMnPO_4_ with cobalt and nickel: A first principle study. Batteries.

[28] Minakshi M., Kandhasamy S. (2012). Utilizing active multiple dopants (Co and Ni) in olivine LiMnPO_4_. Curr. Opin. Solid State Mater. Sci..

[29] Shanmukaraj D., Murugan R. (2004). Synthesis, and characterization of LiNi_y_Co_1−y_PO_4_ (y = 0–1) cathode materials for lithium secondary batteries. Ionics.

[30] Ping L.Z., Jun Z.Y., Ming Z.Y. (2009). Li-site and metal-site ion doping in phosphate-olivine LiCoPO_4_ by first-principles calculation, Chin. Phys. Lett..

[31] Wolfenstine J., Allen J. (2004). LiNiPO_4_–LiCoPO_4_ solid solutions as cathodes. J. Power Sources.

[32] Örnek A., Bulut E., Can M. (2015). Influence of gradual cobalt substitution on lithium nickel phosphate nano-scale composites for high voltage applications. Mater. Charact..

[33] Li Y., Taniguchi I. (2019). Synthesis of LiNi_1-x_Co_x_PO_4_/C nanocomposite cathode for lithium-ion batteries by a combination of aerosol and powder technologies. Adv. Powder Technol..

[34] Rommel S.M., Rothballer J., Schall N., Brünig C., Weihrich R. (2015). Characterization of the carbon-coated LiNi_1−y_ Co_y_PO_4_ solid solution synthesized by a non-aqueous sol-gel route. Ionics.

[35] Neef C., Meyer H.-P., Klingeler R. (2015). Morphology-controlled two-step synthesis and electrochemical studies on hierarchically structured LiCoPO_4_. Solid State Sci..

[36] Murugan A.V., Muraliganth T., Ferreira P.J., Manthiram A. (2009). Dimensionally modulated, single-crystalline LiMPO_4_ (M = Mn, Fe, Co, and Ni) with nano-thumblike shapes for high-power energy storage. Inorg. Chem..

[37] Devaraju M.K., Truong Q.D., Hyodo H., Sasaki Y., Honma I. (2015). Synthesis, characterization, and observation of antisite defects in LiNiPO_4_ nanomaterials. Sci. Rep..

[38] Karthickprabhu S., Hirankumar G., Maheswaran A., Sanjeeviraja C., Bella R.D. (2013). Structural and conductivity studies on LiNiPO_4_ synthesized by the polyol method. J. Alloy. Compd..

[39] Rommel S.M., Schall N., Brünig C., Weihrich R. (2014). Challenges in the synthesis of high voltage electrode materials for lithium-ion batteries: A review on LiNiPO_4_. Monatsh Chem..

[40] Oh S.-M., Myung S.-T., Sun Y.-K. (2012). Olivine LiCoPO_4_-carbon composite showing high rechargeable capacity. J. Mater. Chem..

[41] Wang D., Belharouak I., Ortega L.H., Zhang X., Xu R., Zhou D., Zhou G., Amine K. (2015). Synthesis of high-capacity cathodes for lithium-ion batteries by morphology-tailored hydroxide co-precipitation. J. Power Sources.

[42] Koleva V., Zhecheva E., Stoyanova R. (2010). Ordered olivine-type lithium–cobalt and lithium–nickel phosphates prepared by a new precursor method. Eur. J. Inorg. Chem..

[43] Giorgetti M., Stievano L., Khodaei M., Petaccia L. (2017). X-ray absorption spectroscopy study of battery materials. X-ray Characterization of Nanostructured Energy Materials by Synchrotron Radiation.

[44] Robertson A., Trevino L., Tukamoto H., Irvine J. (1999). New inorganic spinel oxides for use as negative electrode materials in future lithium-ion batteries. J. Power Sources.

[45] Yang C.-C., Hu H.-C., Lin S.J., Chien W.-C. (2014). Electrochemical performance of V-doped spinel Li_4_Ti_5_O_12_/C composite anode in Li-half and Li_4_Ti_5_O_12_/LiFePO_4_-full cell. J. Power Sources.

[46] Guo X., Xiang H.F., Zhou T.P., Li W.H., Wang X.W., Zhou J.X., Yu Y. (2013). Solid-state synthesis, and electrochemical performance of Li_4_Ti_5_O_12_/graphene composite for lithium-ion batteries. Electrochim. Acta.

[47] Ali B., Muhammad R., Islam M., Anang D.A., Han D.-S., Moeez I., Chung K.Y., Cho M.K., Kim J.-Y., Kim M.-G. (2023). Cd-Doped Li_4–x_Cd_x_Ti_5_O_12_ (x = 0.20) as a high rate capable and stable anode material for lithium-ion batteries. ACS Appl. Energy Mater..

[48] Ali B., Muhammad R., Anang D.A., Cho M.K., Kim J.-Y., Nam K.-W. (2020). Ge-doped Li_4_Ti _5-x_Ge_x_O_12_ (x= 0.05) as a fast-charging, long-life bi-functional anode material for lithium-and sodium-ion batteries. Ceram. Int..

[49] Belgibayeva A., Nagashima T., Taniguchi I. (2022). Synthesis of LiCoPO_4_/C nanocomposite fiber mats as free-standing cathode materials for lithium-ion batteries with improved electrochemical properties. Electrochim. Acta.

[50] Alex C., Sarma S.C., . Peter S.C., John N.S. (2020). Competing effect of Co^3+^ reducibility and oxygen-deficient defects toward high oxygen evolution activity in Co_3_O_4_ systems in alkaline medium. ACS Appl. Energy Mater..

[51] Mirghni A.A., Madito M.J., Oyedotun K.O., Masikhwa T.M., Ndiaye N.M., Ray S.J., Manyala N. (2018). A high energy density asymmetric supercapacitor utilizing a nickel phosphate/graphene foam composite as the cathode and carbonized iron cations adsorbed onto polyaniline as the anode. RSC Adv..

[52] Kaus M., Issac I., Heinzmann R., Doyle S., Mangold S., Hahn H., Chakravadhanula V.S.K., Kübel C., Ehrenberg H., Indris S. (2014). Electrochemical delithiation/relithiation of LiCoPO_4_: A two-step reaction mechanism investigated by in situ X-ray diffraction, in situ X-ray absorption spectroscopy, and ex situ ^7^Li/^31^P NMR spectroscopy. J. Phys. Chem. C.

[53] Islam M., Akbar M., Ali G., Nam K.W., Chung K.Y., Jung H.-G. (2020). A high voltage Li-ion full-cell battery with MnCo_2_O_4_/LiCoPO_4_ electrodes. Ceram. Int..

[54] Markevich E., Sharabi R., Gottlieb H., Borgel V., Fridman K., Salitra G., Aurbach D., Semrau G., Schmidt M.A., Schall N. (2012). Reasons for capacity fading of LiCoPO_4_ cathodes in LiPF_6_ containing electrolyte solutions. Electrochem. Commun..

[55] Manzi J., Brutti S. (2016). Surface chemistry on LiCoPO_4_ electrodes in lithium cells: SEI formation and self-discharge. Electrochim. Acta.

[56] Xue W., Huang M., Li Y., Zhu Y.G., Gao R., Xiao X., Zhang W., Li S., Xu G., Yu Y. (2021). Ultra-high-voltage Ni-rich layered cathodes in practical Li metal batteries enabled by a sulfonamide-based electrolyte. Nat. Energy.

[57] Li P., Wang Y., Liu Z., Hu X. (2023). Acid-scavenging separators promise long-term cycling stability of lithium-ion batteries. Mater. Chem. Front..

[58] Örnek A. (2017). Optimization of dielectric heating parameters in the production of high-voltage LiNiPO_4_-core and carbon-shell ceramics. J. Am. Ceram. Soc..

[59] Ni J., Liu W., Liu J., Gao L., Chen J. (2013). Investigation on a 3.2 V LiCoPO_4_/Li_4_Ti_5_O_12_ full battery. Electrochem. Commun..

[60] Du C.Q., Tang Z.Y., Wu J.W., Tang H.Q., Zhang X.H. (2014). A three-volt lithium-ion battery with LiCoPO_4_ and zero-strain Li_4_Ti_5_O_12_ as insertion material. Electrochim. Acta.

[61] Ravel B., Newville M. (2005). ATHENA, ARTEMIS, HEPHAESTUS: Data Analysis for X-Ray Absorption Spectroscopy using IFEFFIT. J. Synchrotron Radiat..

[62] Abbott D.F., Fabbri E., Borlaf M., Bozza F., Schäublin R., Nachtegaal M., Graule T., Schmidt T.J. (2018). Operando X-ray absorption investigations into the role of Fe in the electrochemical stability and oxygen evolution activity of Ni_1-x_Fe_x_O_y_ nanoparticles. J. Mater. Chem. A.

[63] Sharabi R., Markevich E., Borgel V., Salitra G., Gershinsky G., Aurbach D., Semrau G., Schmidt M.A., Schall N., Stinner C. (2012). Raman study of structural stability of LiCoPO_4_ cathodes in LiPF_6_ containing electrolytes. J. Power Sources.

[64] Wu F., Dong J., Chen L., Chen G., Shi Q., Nie Y., Lu Y., Bao L., Li N., Song T. (2023). Removing the intrinsic NiO phase and residual lithium for high-performance nickel-rich materials. Energy Mater. Adv..

