# Supplementary material for: Scalable Precursor-Assisted Synthesis of a High Voltage LiNiyCo1−yPO4 Cathode for Li-Ion Batteries
Source: Nanomaterials (Basel). 2023 Dec 16;13(24):3156. doi: 10.3390/nano13243156 (PMC10746073; doi:10.3390/nano13243156)
Supplement: Supplementary file 1 [file nanomaterials-13-03156-s001.zip › nanomaterials-2738501-supplementary.pdf]

Supplementary Materials

Scalable Precursor-Assisted Synthesis of a High Voltage LiNiyCo1−yPO4 Cathode for Li-Ion Batteries

Mobinul Islam [1], Ghulam Ali [2], Muhammad Faizan [1], Daseul Han [1], Basit Ali [1], Sua Yun [1], Haseeb Ahmad [2] and Kyung-Wan Nam [1,*]

[1] Department of Energy & Materials Engineering, Dongguk University, Seoul 04620, Republic of Korea
[2] U.S.-Pakistan Center for Advanced Studies in Energy, National University of Sciences and Technology (NUST), Islamabad 44000, Pakistan
*Correspondence: knam@dongguk.edu; Tel.: +82-2-2260-4978

Experimental Characterizations

Structural Characterization

The structure of the heat-treated $LiNi_yCo_{1-y}PO_4$ (LNCP) cathode sample was studied using X-ray diffraction (XRD, Rigaku X-ray diffractometer) technique. Field emission-scanning electron microscopy (FE-SEM, JEORJSM-6700F) coupled with energy dispersive spectroscopy (EDS) was used to examine the morphology and elemental mapping. The chemical composition and surface electronic states were investigated by X-ray photoelectron spectrometry (XPS, NEXSA, Thermo Fisher Scientific, USA).

Electrochemical Characterization

LTO electrodes were prepared using 80 wt.% active materials, 10 wt.% carbon black as conducting agent, and 10 wt.% polyvinylidene fluoride (PVDF) in N-methyl-2-pyrrolidone (NMP) as a binder for making the slurry. After continuous mixing, viscous slurries were formed and coated on Al foil. The grahite anodes were prepared by applying similar coditions and were casted on Cu foil. The prepared electrodes were vacuum dried for 12 hours in an oven at 120 °C. The dried electrodes were then roll pressed and punched into disc shapes (diameter: 12 mm, 14 mm).

For the full-cell assembly with the LNCP cathode, an LTO anode or graphte anode was used as a counter electrode, and 1 M $LiPF_6$ in ethylene carbonate and dimethyl carbonate (1:1 volume ratio) with 5 wt.% fluroethylene carbonate was used as an electrolyte. For the full-cell assembly, the active mass of the assembled electrodes was balanced by considering the anode-to-cathode capacity ratio so that the anode mass was controlled to ensure the full utilization of the cathode material. For instance, the active material loadings were controlled at around 1.65 mg/cm^2 for the LTO anode, 0.65 mg/cm^2 for the graphite anode, and 2.8 mg/cm^2 for the LNCP cathode. The anode/cathode mass loading ratio was ~1.0. Biologics VMP-2 multichannel potentiostats and the NEWARE (BT7.6.0) battery testing systems were used for the electrochemical evulation. The cells were cycled at different current rates in the respective voltage window (5.2–3.5 V vs. Li/Li^+ for the LNCP cathode half cell; 1.0–2.5 V vs. Li/Li^+ for the LTO half cell; 3.7–1.5 V vs. Li/Li^+ for the LNCP/LTO full cell; and 5.0–3.5 V vs. Li/Li^+ for the LNCP/graphite full cell) at room temperature (25 °C). The current applied in the full cell was based on the LNCP cathode C-rate (1C = 167 mAh g^{-1}).

Ex situ Characterization

For ex situ XRD and XAS analysis, the cells were disassembled in an argon-filled glove box, then the electrodes were carefully washed with dimethyl carbonate and vacuum sealed in a plastic bag after drying to protect them from exposure to air during transfer to the XRD and XAS chambers. The ex situ XAS measurements of the Co and Ni K-edge were performed at 8C beamlines of the Pohang Accelerations Laboratories (PAL). The energy was selected using a Si(111) double-crystal monochromator (detuned to ~40% of the maximum intensity). Pure cobalt and nickel metallic foils were used as a reference to calibrate the respective spectra. The energy was scanned from -200 eV below to +1000 eV above the respective edges, and the spectra of the electrodes were recorded in the transmission mode. The XAS data were handled and processed using the ATHENA package.

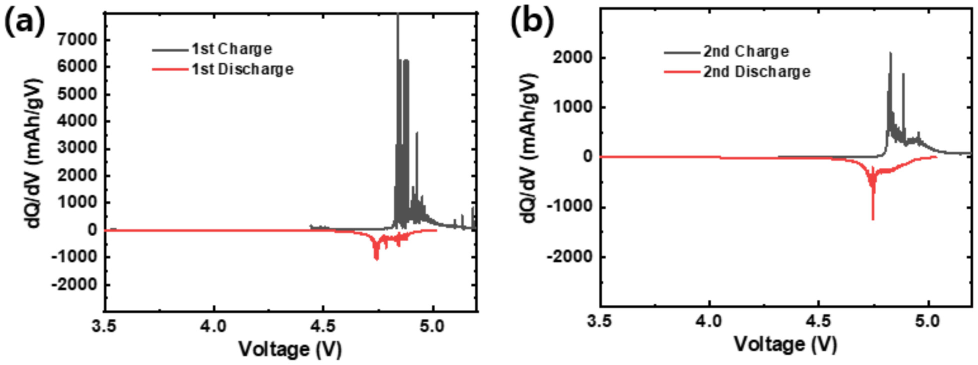

Figure S1. The dQ/dV curves of $LiNi_yCo_{1-y}PO_4$ cathode in a half cell at (a) initial and (b) second cycles.

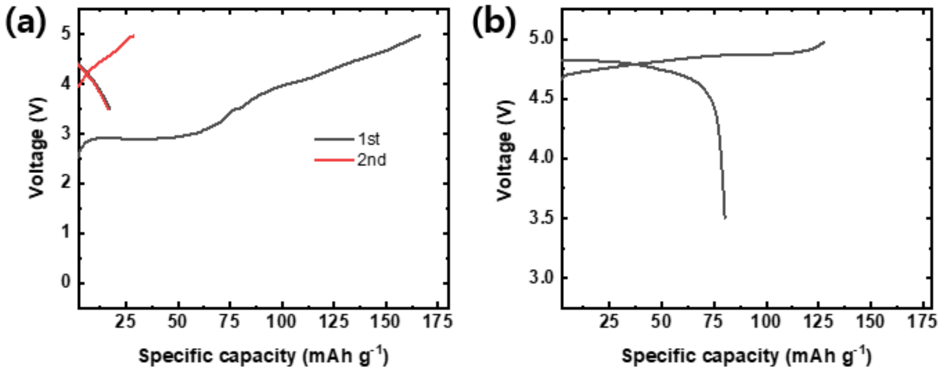

Figure S2. Voltage profile of the LNCP/graphite full cells: (a) without pre-lithiation and (b) with pre-lithiated anode.

A $LiNi_yCo_{1-y}PO_4$/graphite full cell shows a large irreversible charge capacity, which might be due to the extensive electrolyte decomposition associated mainly with SEI layer formation on the graphite anode surface, thus providing a negligible discharge capacity (~17 mA g^{-1}). With a pre-lithiated graphite anode, however, it delivers a high specific discharge capacity (~80 mA g^{-1}) similar to the LNCP half-cell performance. The graphite anode was pre-lithiated by placing them in direct contact with Li foil wetted by the electrolyte solution for 20 minutes.

Table S1. SEM-EDS analysis result for the LNCP cathode.

Element	Weight%	MDL	Atomic%	Error%	Net Int.	R	A	F
O K	45.6	0.19	70.5	8.3	2022.2	0.8809	0.3439	1.0000
P K	17.5	0.17	14.0	4.9	1267.9	0.9162	0.7278	1.0074
Co K	24.6	0.75	10.3	4.9	332.3	0.9642	0.9879	1.0548
Ni K	12.2	0.91	5.1	7.8	132.3	0.9685	0.9891	1.0457
